# Endothelial Dysfunction and Arterial Stiffness in Patients with Inflammatory Bowel Disease: A Systematic Review and Meta-Analysis

**DOI:** 10.3390/jcm11113179

**Published:** 2022-06-02

**Authors:** Hao Wu, Meihua Xu, Hong Hao, Michael A. Hill, Canxia Xu, Zhenguo Liu

**Affiliations:** 1 Center for Precision Medicine and Division of Cardiovascular Medicine, University of Missouri School of Medicine, Columbia, MO 65212, USA; hw5mg@missouri.edu (H.W.); haoho@health.missouri.edu (H.H.); 2 Department of Gastroenterology, Third Xiangya Hospital, Central South University, Changsha 410013, China; 3 Department of Gastroenterology, Xiangya Hospital, Central South University, Changsha 410008, China; meeihuaxu2001@csu.edu.cn; 4 Dalton Cardiovascular Research Center, University of Missouri, Columbia, MO 65212, USA; hillmi@missouri.edu

**Keywords:** inflammatory bowel disease, endothelial dysfunction, arterial stiffness, carotid intima-media thickness, cardiovascular diseases

## Abstract

Population-based studies have suggested that patients with inflammatory bowel disease (IBD) might be at an increased risk for cardiovascular diseases. A meta-analysis was performed on clinical studies to evaluate endothelial function, arterial stiffness, and carotid intima-media thickness (cIMT) in patients with IBD, after searching PubMed, Embase, Cochrane library, and Web of Science databases. A random-effects model was used to allow for the pooling of studies and for determination of the overall effect. After exclusion, a total of 41 eligible studies with 2330 patients with IBD and 2032 matched controls were identified and included for the analysis. It was found that cIMT was significantly increased in patients with IBD as compared with that in matched controls (Cohen’s d: 0.63; 95% CI: 0.34, 0.93; I^2^ = 91.84%). The carotid–femoral pulse wave velocity was significantly higher in patients with IBD compared to that in matched controls (Cohen’s d: 0.76; 95% CI: 0.54, 0.98; I^2^ = 70.03%). The augmentation index was also significantly increased in patients with IBD compared to matched control subjects (Cohen’s d: 0.35; 95% CI: 0.08, 0.63; I^2^ = 61.37%). Brachial artery flow-mediated dilatation was significantly decreased in patients with IBD than that in matched controls (Cohen’s d: −0.73; 95% CI: −1.10, −0.36; I^2^ = 81.02%). Based on the meta-analysis, it was found that patients with IBD exhibit significant endothelial dysfunction, increased arterial stiffness, and cIMT. Thus, patients with IBD may benefit from aggressive risk stratification for cardiovascular diseases.

## 1. Introduction

Cardiovascular diseases (CVDs) remain the leading cause of morbidity and mortality, despite aggressive treatment of traditional risk factors [1]. Endothelial cell dysfunction and systemic inflammation are considered to be among the key factors for the development and progression of CVDs [2,3]. Indeed, patients with chronic inflammatory diseases, such as systemic lupus erythematosus, rheumatoid arthritis, and psoriasis, have an increased risk of arterial and venous thromboembolic events [4]. Population-based studies have also suggested that patients with inflammatory bowel disease (IBD), including ulcerative colitis (UC) and Crohn’s disease (CD), might be at an increased risk for CVDs, including coronary artery disease (CAD) and arterial and venous thromboembolic events [5,6,7,8].

Endothelial dysfunction is critically involved in the development and progression of atherosclerosis and related CAD and stroke. Noninvasive vascular function tests, including flow-mediated dilatation (FMD), nitroglycerin-mediated dilatation (NMD), and reactive hyperemia index (RHI) using peripheral arterial tonometry (PAT), have been used to evaluate in vivo vascular function (either endothelium-dependent or endothelium-independent) [9]. Arterial stiffness is evaluated primarily using pulse wave velocity (PWV) and augmentation index (AIx). Increased arterial stiffness is a well-established indicator for vascular endothelial dysfunction and an independent predictor of cardiovascular events [10].

Atherosclerosis is among the principal contributors to CVDs and is closely associated with endothelial cell dysfunction and increased arterial stiffness [11]. One of the important features of atherosclerosis is smooth muscle hyperplasia with consequential increases in intima-media thickness (IMT), a strong predictor of future cardiovascular events, including myocardial infarction and stroke [12]. Although there are a considerable number of studies and meta-analyses that have evaluated endothelial function, arterial stiffness, and carotid IMT (cIMT) in patients with IBD [13,14,15,16], there is currently no comprehensive and updated study to systematically review, interpret and summarize the data from these individual studies. Thus, the aim of the present study was to perform a systematic review and meta-analysis of studies that evaluated the impact of IBD on endothelial function, arterial stiffness, and cIMT.

## 2. Materials and Methods

The present study was registered with PROSPERO (number CRD42021274093). The corresponding protocol was prospectively developed with defined objectives, specific criteria for study selection, and a detailed approach for critically evaluating the study quality, outcomes, and statistical methods for each study.

### 2.1. Search Strategy

To identify all available studies, a detailed search pertaining to IBD and measurements of in vivo vascular structure and function (i.e., PWV, FMD, cIMT, AIx and RHI) was conducted according to the Preferred Reporting Items for Systematic reviews and Meta-Analyses (PRISMA) guidelines [17]. A systematic search was performed in electronic databases including PubMed, Embase, Cochrane library, and Web of Science, using the following Medical Subject Heading (MeSH) terms and keywords in all possible combinations with Boolean operators: “Inflammatory Bowel Diseases”, “Inflammatory Bowel Disease”, “Bowel Diseases, Inflammatory”, “IBD”, “Ulcerative Colitis”, “Colitis, Ulcerative”, “Idiopathic Proctocolitis”, “Colitis Gravis”, “Inflammatory Bowel Disease, Ulcerative Colitis Type”, “UC”, “Crohn Disease”, “Crohn’s Disease”, “Crohn’s Enteritis”, “CD”, “endothelium-dependent flow-mediated dilatation”, “endothelium-independent flow mediated dilatation”, “FMD”, “nitroglycerin-mediated dilatation”, “NMD”, “endothelial function”, “peripheral arterial tonometry”, “PAT”, “reactive hyperemia”, “vascular stiffness”, “arterial stiffness”, “pulse wave velocity”, “PWV”, “augmentation index”, “AIx”, “intima-media thickness” and “IMT”. The last search was performed on 15th December 2021. In addition, all the references in the retrieved literature were manually reviewed to identify other potentially relevant articles. Animal studies and non-English language articles were excluded. Two independent authors (Hao Wu and Meihua Xu) analyzed each article and performed the data extraction independently. In the case of disagreement, a third investigator was consulted (Canxia Xu or Zhenguo Liu). Any discrepancies were resolved by consensus.

### 2.2. Data Extraction and Quality Assessment

According to the pre-specified protocol, sample size, demographic variables, CV risk factors, total cholesterol (TC), low-density lipoprotein cholesterol (LDLc), high-density lipoprotein cholesterol (HDLc), triglycerides (TGs), disease activity, disease duration, treatment, and quantitative measurements (means with standard deviation) of PWV, FMD, cIMT, AIx, and RHI were extracted from the studies that were included in the present study. Based on the characteristics of the included studies, the methodological quality of each study was evaluated using the Newcastle–Ottawa Scale (NOS), which has been specifically developed to assess the quality of nonrandomized studies in meta-analyses [18].

### 2.3. Statistical Analysis and Risk of Bias Assessment

All statistical analyses were conducted using Stata 17.0 software (StataCorp LLC, College Station, TX, USA). Differences between cases and controls were expressed as Cohen’s d, also known as standardized mean difference, and odds ratios with pertinent 95% confidence intervals (95% CI). A random-effects model was applied to pool the overall effect. Statistical heterogeneity between studies was assessed with Cochran’s Q test and with I^2^ statistic. I^2^ values of 0% indicate no heterogeneity, 25% low, 25–50% moderate, and 50% high heterogeneity [19]. Publication bias was assessed by visual inspection of funnel plots and Egger test. In the case of significant publication bias, nonparametric trim-and-fill analysis was used to obtain an unbiased pooled estimate [20].

### 2.4. Sensitivity Analyses

The sensitivity analyses were re-examined by only including the studies judged as “high quality” according to NOS (i.e., NOS ≥ 7). In addition, considering the potential difference between pediatric IBD and adult IBD, a further analysis was performed after excluding the studies examining pediatric patients. Finally, for the studies with pre-existing CV risk factors that were not excluded or specifically described, a sensitivity analysis was conducted after excluding the studies with such pre-existing CV risk factors.

### 2.5. Subgroup Analyses

Considering the potential influence of the types of IBD on the analysis outcomes, separate subgroup analyses were performed for the studies on CD and UC, respectively.

### 2.6. Meta Regression Analyses

We hypothesized that differences among the included studies may be affected by clinical variables including disease activity, disease duration and treatment. To explore the effect of such variables on predicting Cohen’s d, a meta-regression analysis was conducted with the restricted maximum likelihood (ReML) estimation method. Meta-regression was not considered when there were fewer than ten studies in a meta-analysis according to the Cochrane Handbook [21].

## 3. Results

### 3.1. Descriptions of the Identified Studies

**Figure 1:**
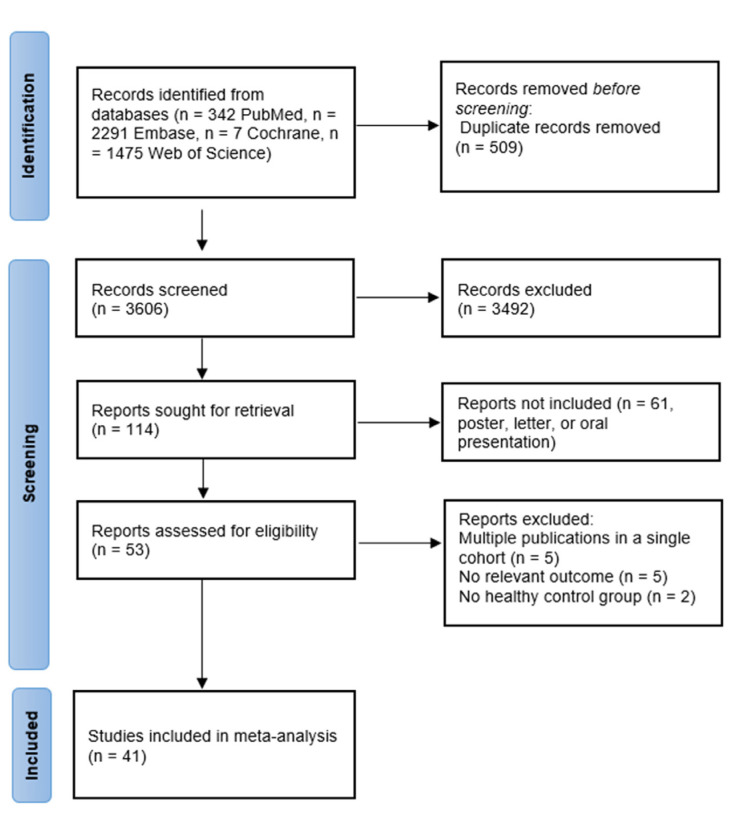
PRISMA flow diagram for identification of studies for meta-analysis. PRISMA, Preferred Reporting Items for Systematic reviews and Meta-Analyses.

After excluding duplicate studies, a total of 3606 articles were retrieved from the database search. Of these studies, 3492 were excluded because they did not meet the inclusion criteria for the present study after review. In addition, the following were excluded: 61 posters, letters, or oral presentations, 5 multiple publications in a single cohort, 5 without relevant outcomes, and 2 without healthy controls. Thus, 41 studies with 2330 IBD patients and 2032 control subjects were included in the final analysis (Figure 1).

### 3.2. Study Characteristics

The primary characteristics of the studies and NOS are summarized in Table 1 and Table S1. The data on disease activity and ongoing therapy for IBD are listed in Table S2. The measurements of endothelial function, arterial stiffness, and IMT are shown in Table S3. Of note, two studies showed that the levels of aortic PWV were significantly higher in patients with IBD than in controls [22,23]. However, another study reported that the brachial-ankle PWV was not significantly different between the patients with IBD and the controls [24]. In three studies on brachial artery NMD, two studies revealed that NMD was significantly lower in patients with IBD than in controls [25,26], whereas the other showed that there was no difference in NMD between IBD patients and controls [27]. In the included studies, the mean age was 37.4 years for patients with IBD and 37.7 years for matched controls (*p* = 0.06). The percentage of females was 45.4% in patients with IBD and 48.6% in matched controls (*p* = 0.11), indicating that the patients and controls were well age- and sex-matched. Smoking was reported in 0–35.8% of patients, hypertension in 0–38.1%, and diabetes mellitus (DM) in 0–10% (Table S1). There were no differences in the prevalence of smoking, hypertension, and DM between IBD patients and matched controls. IBD patients had a significantly lower BMI compared with the controls. In regard to lipid profile, patients with IBD had significantly lower levels of TC, LDLc, and HDLc compared with the controls, without a difference in the levels of TGs (Table 1).

### 3.3. Carotid Intima-Media Thickness Was Significantly Increased in IBD Patients

**Figure 2:**
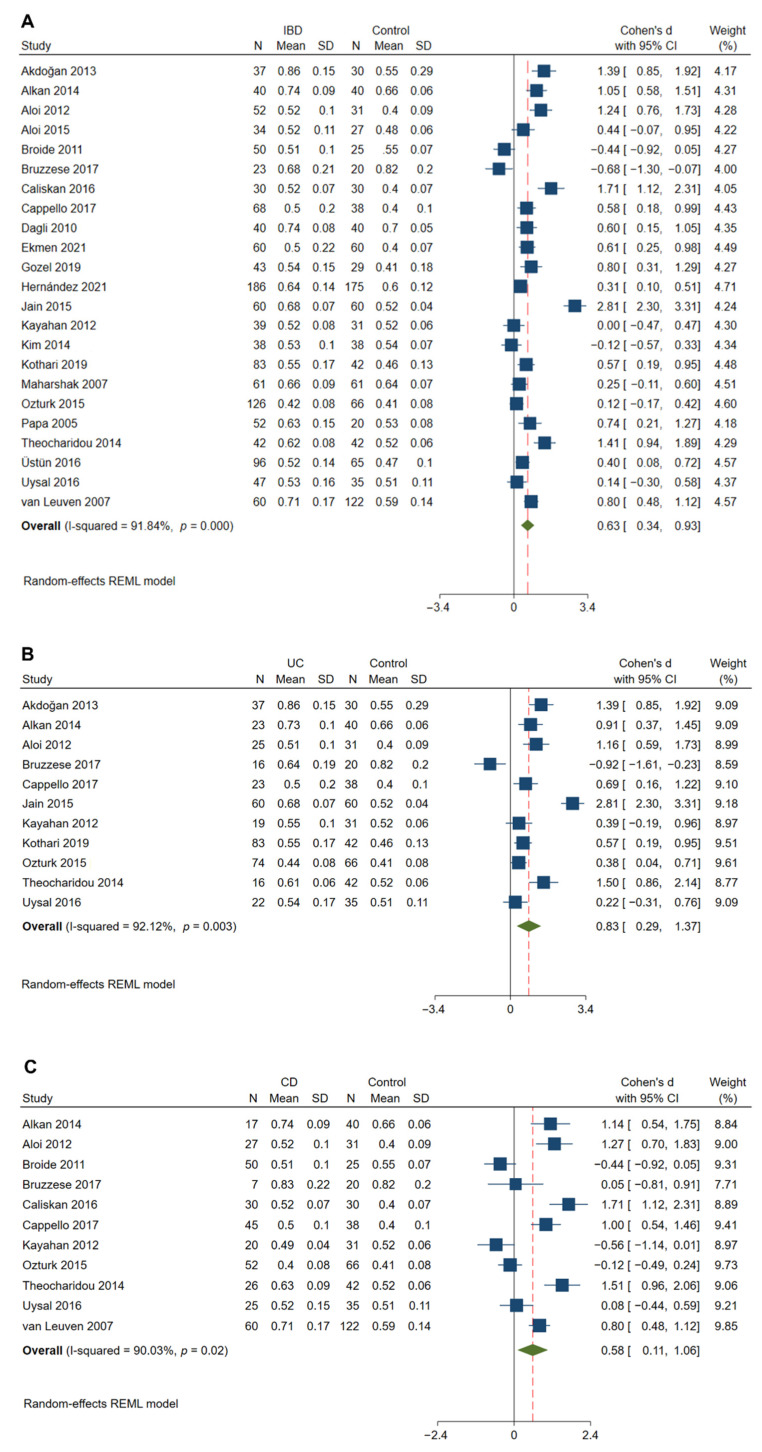
Meta-analysis of studies reporting on cIMT in patients with IBD compared to matched controls. Comparisons include overall IBD vs. control (**A**), UC vs. control (**B**), and CD vs. control (**C**). cIMT, carotid intima-media thickness; IBD, inflammatory bowel disease; UC, ulcerative colitis; CD, Crohn’s disease; REML, restricted maximum likelihood.

In 23 studies with 1367 IBD patients and 1127 matched controls [13,25,27,28,29,30,31,32,33,34,35,36,37,38,39,40,41,42,43,44,45,46,47], cIMT was found to be significantly increased in patients with IBD compared with the matched controls (Cohen’s d: 0.63; 95% CI: 0.34, 0.93; *p* < 0.01, Figure 2A). However, the heterogeneity among the studies was significant (I^2^ = 91.84%; *p* < 0.01).

### 3.4. Arterial Stiffness Was Significantly Increased in IBD Patients

**Figure 3:**
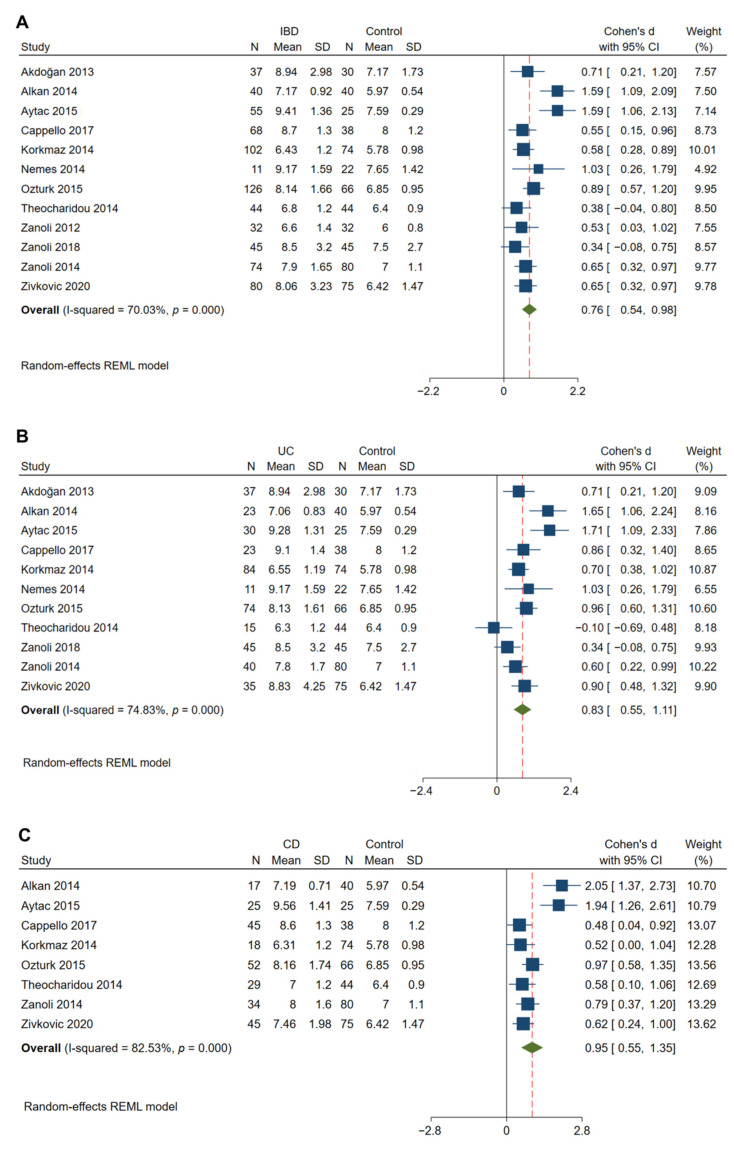
Meta-analysis of studies reporting on cfPWV in patients with IBD compared to matched controls. Comparisons include overall IBD vs. control (**A**), UC vs. control (**B**), and CD vs. control (**C**). cfPWV, carotid-femoral pulse wave velocity.

**Figure 4:**
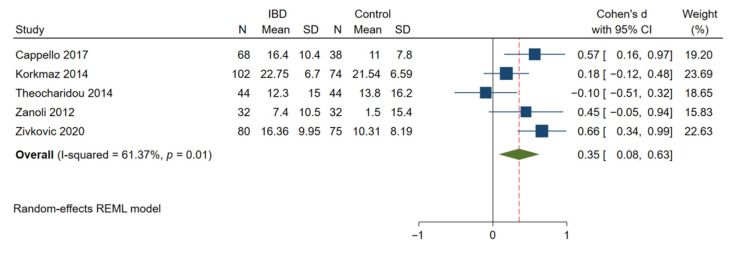
Meta-analysis of studies reporting on AIx in patients with IBD compared to matched controls. AIx, augmentation index.

Twelve studies that evaluated 714 patients with IBD and 571 matched controls showed that a significantly higher cfPWV (indicative of arterial stiffening) was observed in IBD patients compared to that in control subjects (Cohen’s d: 0.76; 95% CI: 0.54, 0.98; *p* < 0.01, Figure 3A) [13,28,29,35,48,49,50,51,52,53,54,55]. Significant heterogeneity was observed to be present among the studies (I^2^ = 70.03%; *p* < 0.01). In 5 studies with 326 patients with IBD and 263 matched controls [35,49,51,52,55], a significantly higher AIx was found in the IBD patients compared to controls (Cohen’s d: 0.35; 95% CI: 0.08, 0.63; *p* = 0.01, Figure 4). The heterogeneity among these studies was significant with an I^2^ of 61.37% (*p* = 0.03).

### 3.5. Endothelial Function Was Significantly Impaired in Patients with IBD

**Figure 5:**
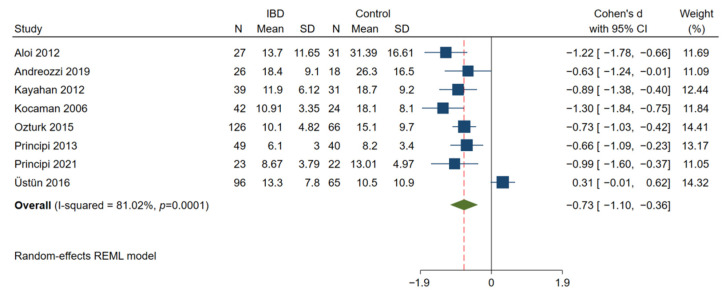
Meta-analysis of studies reporting on FMD in patients with IBD compared to matched controls. FMD, flow-mediated dilatation.

**Figure 6:**
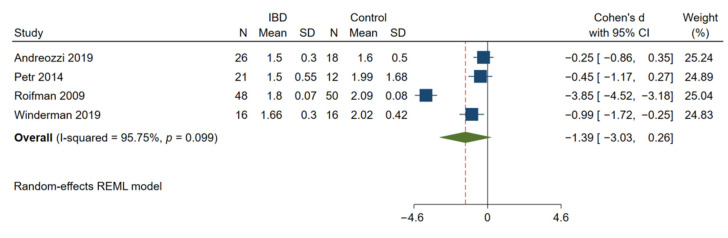
Meta-analysis of studies reporting on RHI in patients with IBD compared to matched controls. RHI, reactive hyperemia index.

In 8 studies with 428 IBD patients and 297 matched control subjects [13,14,25,26,27,30,56,57], a significant reduction in brachial artery FMD was observed in patients with IBD as compared with the controls (Cohen’s d: −0.73; 95% CI: −1.10, −0.36; *p* < 0.01, Figure 5). There was significant heterogeneity among these studies (I^2^ = 81.02%; *p* < 0.01). However, there was no difference in the measured RHI between 111 patients with IBD and 96 matched controls (Cohen’s d: −1.39; 95% CI: −3.03, 0.26; *p* = 0.10, Figure 6) [56,58,59,60].

### 3.6. Publication Bias

To assess publication bias, a visual inspection of funnel plots of effect size versus standard error for studies evaluating cIMT and cfPWV demonstrated a symmetrical shape, suggesting that there was no significant publication bias (Figure S1A,B). In addition, the Egger test confirmed that there was no statistically significant publication bias. However, the funnel plot for studies evaluating branchial artery FMD showed an asymmetrical shape. Thus, the nonparametric trim-and-fill analysis of publication bias was conducted. The corrected Cohen’s d was −0.50 (95% CI: −0.86, −0.15) (from −0.73; 95% CI: −1.10, −0.36) when the observed studies were combined with three imputed studies (Figure S1C,D), suggesting that the publication bias of the observed studies was not from the studies per se, and, thus, would not compromise the interpretation of the data from these studies.

### 3.7. Sensitivity Analyses

The studies considered as “high quality” (NOS ≥ 7) were included in the analysis (Table 2, Panel A). In 17 studies with 1023 IBD patients and 905 matched control subjects, cIMT was found to be significantly increased in patients with IBD compared with the matched controls (Cohen’s d: 0.71; 95% CI: 0.35, 1.08; *p* < 0.01). In 10 studies with 648 IBD patients and 524 matched control subjects, cfPWV was found to be significantly increased in patients with IBD compared with the matched controls (Cohen’s d: 0.67; 95% CI: 0.49, 0.86; *p* < 0.01). In 3 studies with 214 IBD patients and 137 matched control subjects, a significant reduction in brachial artery FMD was observed in patients with IBD as compared with the controls (Cohen’s d: −0.74; 95% CI: −0.96, −0.52; *p* < 0.01). Similarly, except for the studies with pediatric patients, the results on cIMT, cfPWV, and FMD were confirmed (Table 2, Panel B). Further, repeated analyses also confirmed the results on cIMT and cfPWV when the studies with specific data on patients with IBD and matched controls without traditional CV risk factors were included in the analysis (Table 2, Panel C).

### 3.8. Subgroup Analyses

Considering the potential impact of the IBD type (UC and CD) on the outcomes, separate subgroup analyses were performed. Similarly, cIMT was found to be significantly increased in patients with UC (Cohen’s d: 0.83; 95% CI: 0.29, 1.37; *p* < 0.01) and CD (Cohen’s d: 0.58; 95% CI: 0.11, 1.06; *p* = 0.02) compared with the matched controls (Figure 2B,C). A significantly higher cfPWV was observed in both UC (Cohen’s d: 0.83; 95% CI: 0.55, 1.11; *p* < 0.01) and CD (Cohen’s d: 0.95; 95% CI: 0.55, 1.35; *p* < 0.01) patients compared to that in control subjects (Figure 3B,C)

### 3.9. Meta-Regression Analyses

Meta-regression was performed to determine the sources of heterogeneity and assess the impact of the variables (disease activity, disease duration and treatment) on cIMT. The regression model showed that these clinical variables did not have a significant impact on the association between IBD and cIMT (Table 3). No meta-regression analysis was performed for PWV, FMD, and AIx because of the limited number of studies.

## 4. Discussion

The data from the present meta-analysis have shown that IBD is associated with significant endothelial dysfunction with impaired FMD, increased arterial stiffness, and increased cIMT. Importantly, the findings on impaired FMD, increased cfPWV, and cIMT in patients with IBD were confirmed using appropriate sensitivity and subgroup analyses. In contrast, no significant association between IBD and RHI was identified. Regression models demonstrated that disease activity, disease duration or treatment might not have a significant impact on cIMT in patients with IBD. Of note, the data also showed that significantly lower serum levels of TC, LDLc, and HDLc were observed in patients with IBD than in controls. The results of the present meta-analysis are consistent with previous published work [15,16]. Further, we found a significantly higher AIx in the IBD patients compared to controls, and traditional CV risk factors (i.e., lipid profile) may not significantly contribute to the outcomes of endothelial function, arterial stiffness, and cIMT in the present study.

Endothelial dysfunction is critically involved in the development and progression of atherosclerosis-related vascular diseases, including CAD, PAD, and stroke [61]. Microvascular endothelial function can be measured in vivo with RHI using PAT, whereas macrovascular endothelial function can be evaluated with brachial artery FMD, and endothelium-independent vascular function can be evaluated with NMD. The present study showed that FMD was significantly impaired in patients with IBD compared to controls. FMD is an endothelium-dependent vascular function with endothelium-derived nitric oxide (NO) as one of the principal mediators [9], and has been widely used as a surrogate marker of vascular disease and a predictor of incident CV events in human subjects [62]. NMD, endothelium-independent vasodilation, assessed by the sublingual administration of nitroglycerine, has been used to determine if there is an impairment in vasodilation due to vascular smooth muscle cell dysfunction, or the inability of NO to be released into the vasculature. NMD has been suggested to serve as a marker of CVDs such as atherosclerosis in human subjects with and without cardiovascular risk factors [63]. RHI does not directly measure vasodilation, rather, the augmentation of finger pressure, which is believed to reflect microvascular dilation through both endothelium-dependent and -independent mechanisms [64]. Clinical studies have reported that RHI is significantly lower in patients with coronary endothelial dysfunction than in normal controls [65]. However, the present study demonstrated that IBD patients appear to have preserved RHI compared with controls.

The present study showed that cfPWV and aortic AIx were significantly increased in patients with IBD compared to the controls. Several studies have demonstrated that cfPWV or aortic PWV is a strong predictor of future CV events and all-cause mortality in various populations [10,66,67]. A meta-analysis has similarly reported that AIx is an independent predictor of future CV events and all-cause mortality [68]. A general population-based comparative study has shown that a high AIx predicts an increased rate of mortality and CV events in men, but not in women [69]. One of the important features of arterial atherosclerosis is smooth muscle hyperplasia with increased cIMT, a strong predictor of future vascular events, including myocardial infarction and stroke [12]. The present study demonstrated that cIMT was significantly increased in patients with IBD compared with the matched controls, which was confirmed with appropriate sensitivity and subgroup analyses.

In the present study, no differences in traditional CV risk factors, including age, sex, smoking, hypertension, and DM, were observed between patients with IBD and matched controls. Interestingly, the data on lipid profiles showed that lower levels of TC, LDLc, and HDLc were observed in patients with IBD than in controls. Thus, these traditional CV risk factors may not significantly contribute to the outcomes of endothelial function, arterial stiffness, and cIMT in the present study. Since most of the included studies specifically enrolled IBD patients and controls without traditional CV risk factors, the present study could not determine the prevalence of the traditional CV risk factors among IBD patient populations. Two population studies reported a higher prevalence of DM and hypertension in IBD patients [70,71]. However, another study in the UK demonstrated that the prevalence of obesity, hypertension and hyperlipidemia was lower in IBD patients than matched controls; however, the proportion of smokers was higher in the patients with CD than in those with UC and in controls [72]. In contrast, a Danish study showed that the prevalence estimates of hypertension and DM were not significantly different between IBD patients and the control population [73]. Thus, the data on the prevalence of traditional CV risk factors have been inconsistent from different population-based studies in IBD patients.

IBD is a chronic pathological condition with significant local and systemic non-infectious inflammation. Several inflammatory biomarkers, including CRP, TNF-α, IL-1β, and IL-6, are significantly increased in patients with IBD. A study with over 100,000 subjects has shown a significant increase in the serum CRP level in IBD patients with an increased risk for CAD [5,74]. Increased levels of TNF-α and IL-6, through increased production of reactive oxygen species, may lead to endothelial dysfunction with an increased risk of CVDs and less optimal overall outcomes [75,76]. A multicenter longitudinal study has demonstrated that active disease and disease duration are associated with aortic stiffening in IBD patients [22]. Interestingly, the meta-regression analysis in the present study showed that CRP and ESR levels, disease duration and treatment might not have a significant impact on cIMT in IBD patients, which may relate to significant heterogeneity among the selected studies. Thus, prospective investigations are needed to make precise estimates. It is also important to consider that medications that decrease inflammatory burden in IBD patients may, in turn, also lead to a decreased risk for CVDs. However, data on the potential effects of salicylates, for example, on CVDs are inconsistent. A Danish study showed that the risk of CAD was lower in IBD patients using salicylates than in non-users [74]. In contrast, a UK cohort revealed opposite results, with a higher risk of CVDs in IBD patients receiving salicylates [77]. Systemic use of steroids could be detrimental with an increased risk of CVDs and associated metabolic abnormalities [78]. Compared to salicylates and steroids, anti-TNF-α therapy substantially reduces disease activity in IBD patients and is consistently associated with a decreased risk of CVDs [22,79,80]. The latest international consensus on the prevention of venous and arterial thrombotic events in patients with IBD proposed that exposure to steroids should be limited, and anti-TNF-α therapy might be associated with a reduced risk of thrombotic events [81].

There were several limitations in the present study. First, most of the included studies were cross-sectional in design. Second, there was significant heterogeneity among studies, likely due to different inclusion and exclusion criteria, different status of disease activity and treatment (i.e., new-onset, remission, and flare), and diverse study methodology. Thirdly, most matched controls were volunteers or hospital controls, and some of the studies did not specifically report the status of traditional CV risk factors in IBD patients and their controls. Finally, the potential impact of medications in IBD patients on CV outcomes was not fully analyzed, since most of the included studies only provided information on medications and did not analyze the relationship between medications and CV outcomes in IBD patients.

## 5. Conclusions

In conclusion, the present meta-analysis showed that IBD was significantly associated with endothelial dysfunction, increased arterial stiffness, and cIMT. Thus, patients with IBD may benefit from aggressive risk stratification for CVDs.

## Figures and Tables

**Table 1:** Estimated risks and differences in clinical variables between IBD subjects and controls.

Variable	Population (n) IBD/Controls	Studies (n)	IBD	Controls	Log Odds Ratio or Cohen’s d	95% CI	*p* Value	I^2^ %
Female	1058/988	41	45.4%	48.6%	−0.100	−0.223, 0.023	0.1127	0
Smoking	241/195	35	12.5%	11.6%	0.090	−0.131, 0.312	0.4252	0
Hypertension	71/66	33	3.5%	3.8%	0.054	−0.292, 0.400	0.7586	0
DM	15/16	39	0.7%	0.8%	−0.095	−0.601, 0.412	0.7134	0
Age (years)	2330/2032	41	37.4	37.7	0.081	−0.003, 0.165	0.06	43.46
BMI (kg/m^2^)	2136/1862	36	23.9	24.7	−0.237	−0.369, −0.105	0.0004	74.71
TC (mg/dL)	1549/1368	26	172	184	−0.238	−0.365, −0.111	0.0002	61.94
LDLc (mg/dL)	1486/1238	25	102	111	−0.200	−0.349, −0.050	0.0088	70.94
HDLc (mg/dL)	1557/1340	25	50	52	−0.152	−0.267, −0.037	0.0095	52.88
TGs (mg/dL)	1638/1360	26	114	112	−0.042	−0.175, 0.091	0.5368	66.00

IBD, inflammatory bowel disease; DM, diabetes mellitus; BMI, body mass index; TC, total cholesterol; LDLc, low-density lipoprotein cholesterol; HDLc, high-density lipoprotein cholesterol; TGs, triglycerides.

**Table 2:** Sensitivity analyses. Panel A: “high quality” studies (Newcastle–Ottawa Scale ≥ 7) included; Panel B: exclusion of studies reporting on the pediatric IBD; Panel C: inclusion of studies specifically reporting on patients with IBD and matched controls without traditional cardiovascular risk factors.

	No. of Studies	No. of Cases/Controls	Cohen’s d [95% CI]	*p* Value	I^2^ %
**Panel A**					
cIMT	17	1023/905	0.710 [0.345, 1.075]	0.0001	92.89
cfPWV	10	648/524	0.673 [0.488, 0.857]	<0.0001	55.99
FMD	3	214/137	−0.741 [−0.964, −0.519]	<0.0001	0
**Panel B**					
cIMT	21	1281/1069	0.616 [0.295, 0.937]	0.0002	92.55
cfPWV	12	714/571	0.759 [0.541, 0.977]	<0.0001	70.03
FMD	6	375/248	−0.679 [−1.134, −0.225]	0.0034	85.42
**Panel C**					
cIMT	11	506/379	0.652 [0.073, 1.231]	0.0272	93.74
cfPWV	6	348/296	0.858 [0.426, 1.290]	0.0001	84.80
FMD	0	NA	NA	NA	NA

IBD, inflammatory bowel disease; cIMT, carotid intima-media thickness; cfPWV, carotid-femoral pulse wave velocity; FMD, flow-mediated dilatation.

**Table 3:** Random-effects meta-regression of cIMT.

Moderators	Coefficient	95% CI	Z-Test	*p* Value
CRP	−0.0097	−0.0441, 0.0247	−0.55	0.580
ESR	0.0265	−0.0053, 0.0584	1.63	0.102
Disease duration	−0.0462	−0.1544, 0.0621	−0.84	0.403
Salicylates	0.0033	−0.0085, 0.0151	0.54	0.488
Steroids	−0.0130	−0.0304, 0.0045	−1.46	0.145
Immunomodulator	−0.0047	−0.0244, 0.0150	−0.47	0.640
Biologics	−0.0104	−0.0233, 0.0025	−1.58	0.113

cIMT, carotid intima-media thickness; CRP, C-reactive protein; ESR, erythrocyte sedimentation rate.

## Data Availability

Not applicable.

## Supplementary Materials

The following supporting information can be downloaded at: https://www.mdpi.com/article/10.3390/jcm11113179/s1, Table S1: Participant characteristics of included studies; Table S2: Measures of disease activity and data on treatment in patients with IBD of included studies; Table S3: Outcomes of patients with IBD and Controls in included studies; Figure S1: Funnel plot of publication bias of cIMT, cfPWV, and FMD. References [13,14,22,23,24,25,26,27,28,29,30,31,32,33,34,35,36,37,38,39,40,41,42,43,44,45,46,47,48,49,50,51,52,53,54,55,56,57,58,59,60] are cited in the supplementary materials.
