# Supplementary material for: Endothelial Dysfunction and Arterial Stiffness in Patients with Inflammatory Bowel Disease: A Systematic Review and Meta-Analysis
Source: J Clin Med. 2022 Jun 2;11(11):3179. doi: 10.3390/jcm11113179 (PMC9181134; doi:10.3390/jcm11113179)
Supplement: Supplementary file 1 [file jcm-11-03179-s001.zip › jcm-1743017-supplementary.pdf]

Table S1. Participant characteristics of included studies.

Author	NOS		Pop (n)	M/F	Age (years)	HTN (%)	Smoking (%)	DM (%)	Ob (%)	BMI (kg/m^2)	TC (mg/dl)	LDLc (mg/dl)	HDLc (mg/dl)	TGs (mg/dl)
Akdoğan (28)	8	UC	37	21/16	48	16	16	5	NA	27.0	196	135	45	135
		Controls	30	9/21	45	13	13	7	NA	27.5	198	126	45	135
Alkan (29)	8	IBD	40	22/18	38.4	0	0	0	NA	23.7	178	108	52	109
		Controls	40	22/18	38.3	0	0	0	NA	23.7	170	99	50	117
Aloi (30)	6	IBD	52	23/29	15.2	NA	18.8	0	NA	NA	NA	NA	NA	NA
		Controls	31	12/19	11.9	NA	0	0	NA	NA	NA	NA	NA	NA
Aloi (31)	7	IBD	34	20/14	11.1	NA	6	0	0	18.9	147	89	58	71
		Controls	27	19/8	11.3	NA	0	0	0	24.3	145	66	87	71
Andreozzi (56)	6	IBD	26	10/16	13.3	NA	NA	NA	NA	17.1	135	71	49	NA
		Controls	18	10/8	12.5	NA	NA	NA	NA	18.9	141	72	51	NA
Aytac (48)	5	UC	30	15/15	44.7	0	NA	0	0	24.9	177	107	48	80
		CD	25	12/13	38.9	0	NA	0	0	23	155	100	44	90
		Controls	25	12/13	42.1	0	NA	0	NA	25.4	169	97	46	136
Broide (32)	8	CD	50	28/22	31.5	0	NA	0	NA	22.6	NA	79	54	114
		Controls	25	13/12	32	0	NA	0	NA	24.1	NA	102	61	105
Bruzzese (33)	6	IBD	23	17/6	49.4	0	0	0	0	NA	NA	NA	NA	NA
		Controls	20	10/10	51	0	0	0	0	NA	NA	NA	NA	NA
Caliskan (34)	7	CD	30	16/14	41	NA	0	0	NA	24.3	195	120	58	110

		Controls	30	14/16	42	NA	0	0	NA	24.7	201	120	54	132
Cappello (35)	7	IBD	68	35/33	31.6	4.4	35.8	2.9	NA	22.5	160	90	52	93
		Controls	38	18/20	30.4	2.6	31.6	2.6	NA	23.9	177	104	57	81
Dagli (36)	8	IBD	40	23/17	39.4	0	0	0	0	24.6	182	119	46	137
		Controls	40	22/18	38.1	0	0	0	0	25.8	186	117	52	114
Ekmen (37)	8	IBD	60	35/25	36.9	0	31.7	0	NA	24.4	NA	NA	NA	NA
		Controls	60	34/26	36.9	0	35	0	NA	26.0	NA	NA	NA	NA
Fan (24)	7	IBD	42	19/23	50	NA	20	10	NA	26.9	202	123	57	89
		Controls	73	34/39	51	NA	4	10	NA	25.6	211	133	58	80
Gozel (38)	5	IBD	43	26/17	31.4	0	0	0	NA	23.7	NA	NA	NA	NA
		Controls	29	12/17	31.3	0	0	0	NA	24.3	NA	NA	NA	NA
Hernández (39)	7	IBD	186	85/101	48	17	19	0	27	27	204	117	57	147
		Controls	175	89/86	45	11	18	0	13	26	200	120	58	103
Jain (40)	7	UC	60	33/27	35.6	0	0	0	0	20.4	137	80	40	88
		Controls	60	32/28	34.9	0	0	0	0	21	137	79	40	88
Kayahan (25)	7	IBD	39	21/18	34.4	0	5	0	NA	22.5	162	88	53	104
		Controls	31	16/15	32.7	0	9	0	NA	25.3	171	98	53	98
Kim (41)	7	IBD	38	26/12	38.5	0	0	0	0	21.1	173	89	54	117
		Controls	38	26/12	38.7	0	0	0	0	24.1	198	115	60	125
Kocaman (26)	5	UC	42	16/26	37.9	0	14.3	0	NA	25	185	NA	NA	110

		Controls	24	8/16	38.1	0	12.5	0	NA	24.7	180	NA	NA	114
Korkmaz (49)	8	IBD	102	66/36	43.9	0	0	0	0	26.1	164	117	43	136
		Controls	74	46/28	44.6	0	0	0	0	26.8	194	131	39	144
Kothari (42)	6	UC	83	47/36	37.1	0	0	0	NA	18.8	137	92	40	99
		Controls	42	25/17	37.2	0	0	0	NA	23.6	136	91	41	103
Maharshak (43)	8	IBD	61	28/33	36	8	15	1.6	NA	23.1	NA	NA	NA	NA
		Controls	61	28/33	36	15	10	1.6	NA	23.1	NA	NA	NA	NA
Nemes (50)	6	UC	11	7/4	39	9	18	0	NA	27.4	NA	NA	NA	138
		Controls	22	11/11	38.8	9	23	0	NA	26.4	NA	NA	NA	132
Ozturk (13)	7	IBD	126	97/29	31.7	0	19.8	0	NA	23.7	NA	99	46	112
		Controls	66	45/21	30.9	0	16.7	0	NA	23.6	NA	104	50	123
Papa (44)	8	IBD	52	34/18	34	0	0	0	NA	23.5	141	83	42	113
		Controls	20	13/7	32	0	0	0	NA	24.1	144	86	44	112
Petr (58)	6	CD	21	15/6	14.8	NA	0	NA	NA	19	152	79	55	104
		Controls	12	2/10	16.2	NA	0	NA	NA	20.8	172	97	61	100
Principi (14)	7	IBD	49	25/24	41	14	NA	8	NA	24.2	166	96	47	116
		Controls	40	16/24	45	10	NA	10	NA	25.3	171	100	49	112
Principi (57)	6	IBD	23	9/14	51.2	38.1	21.7	8.7	NA	24.4	NA	NA	NA	NA
		Controls	22	12/10	48.6	40.9	4.5	4.5	NA	23	NA	NA	NA	NA
Roifman (59)	7	IBD	48	26/22	39.2	6.3	10.4	0	NA	24.7	NA	NA	NA	NA

		Controls	50	22/28	37	2	2	0	NA	24.9	NA	NA	NA	NA
Theocharidou (45)	8	IBD	42	20/22	36.1	2.4	52.4	0	NA	23.8	175	107	48	94
		Controls	42	20/22	36.3	11.9	52.4	0	NA	23.2	194	125	49	98
Theocharidou (51)	8	IBD	44	22/22	36.1	2.3	40.9	0	NA	23.7	174	106	49	88
		Controls	44	22/22	37.2	9.1	40.9	0	NA	24.3	201	129	51	98
Üstün (27)	6	IBD	96	41/55	43.7	0	10.4	0	NA	24.8	185	105	NA	134
		Controls	65	22/43	41.2	0	26.6	0	NA	25.6	190	110	NA	132
Uysal (46)	6	IBD	47	26/21	41.9	0	0	0	NA	23.7	NA	NA	NA	NA
		Controls	35	19/16	41.4	0	0	0	NA	24	NA	NA	NA	NA
van Leuven (47)	7	CD	60	27/33	42.4	8.3	21.7	0	NA	24.4	176	100	59	82
		Controls	122	55/67	40.5	5.7	18	0	NA	25	195	116	57	119
Winderman (60)	8	IBD	16	7/9	16.7	0	0	0	NA	23.5	163	96	54	74
		Controls	16	7/9	15.1	0	0	0	NA	23.2	159	85	53	85
Zanoli (52)	8	IBD	32	19/13	30	0	0	0	0	23.5	162	95	46	NA
		Controls	32	19/13	31	0	0	0	0	24.3	NA	NA	NA	NA
Zanoli (53)	8	UC	45	26/19	48	NA	0	0	NA	24	170	95	54	90
		Controls	45	26/19	44	NA	0	0	NA	26	182	107	46	84
Zanoli (22)	9	UC	82	45/37	37	0	NA	0	NA	24	158	NA	48	102
		CD	85	52/33	39	0	NA	0	NA	24	173	NA	50	128
		Controls	167	95/72	38	0	NA	0	NA	25	176	NA	50	119

Zanoli (54)	8	IBD	74	37/37	37	NA	NA	0	NA	24.2	160	NA	NA	NA
		Controls	80	40/40	38	NA	NA	0	NA	24.7	NA	NA	NA	NA
Zanoli (23)	8	CD	86	43/43	40	0	0	0	NA	24.1	NA	NA	NA	NA
		Controls	86	43/43	40	0	0	0	NA	24.7	NA	NA	NA	NA
Zivkovic (55)	7	IBD	80	50/30	39.8	0	23.7	0	2.5	24	186	109	51	122
		Controls	75	46/29	38.5	0	16.4	0	4	24.7	204	127	55	107

NOS, Newcastle-Ottawa Scale; HTN, hypertension; DM, diabetes mellitus; Ob, obesity; UC, ulcerative colitis; CD, Crohn’s disease; IBD, inflammatory bowel disease.

Table S2. Measures of disease activity and data on treatment in patients with IBD of included studies.

Author	Pop (n)	CRP mg/L	ESR mm/h	Disease duration (years)	Salicylates %	Steroids %	IM %	Biologics %
Akdoğan (28)	UC (37)	8.3	22.4	NA	100	4	11	5
Alkan (29)	IBD (40)	4.4	21.4	4.35	100	2.5	7.5	0
Aloi (30)	IBD (52)	13.58	50.78	3.97	75	35	46	19
Aloi (31)	IBD (34)	17.47	31.1	0.94	NA	29	50	35
Andreozzi (56)	IBD (26)	14.5	26.5	NA	42.9	21.4	7.1	NA
Aytac (48)	UC (30)	8.17	19.25	NA	NA	NA	NA	NA
	CD (25)	19.08	23.65	NA	NA	NA	NA	NA
Broide (32)	CD (50)	14.6	NA	10.9	60	54	38	30
Bruzzese (33)	IBD (23)	NA	NA	11.7	NA	NA	NA	100
Caliskan (34)	CD (30)	1.07	NA	NA	NA	NA	NA	NA
Cappello (35)	IBD (68)	8.4	14.3	NA	72.1	26.5	33.8	22.1
Dagli (36)	IBD (40)	13	32	4.16	97.5	NA	NA	2.5
Ekmen (37)	IBD (60)	1	11.5	4	58.3	50	41.7	NA
Fan (24)	IBD (42)	1.7	NA	11.5	48	16.7	40	14
Gozel (38)	IBD (43)	23.9	22.7	1.4	100	7	16.3	9.3
Hernández (39)	IBD (186)	1.8	NA	12	32	3	42.5	30

Jain (40)	UC (60)	NA	41.06	7.24	NA	NA	NA	NA
Kayahan (25)	IBD (39)	7.8	25.5	5.03	69.2	12.8	10.3	2.6
Kim (41)	IBD (38)	6.78	29	4.33	100	26.3	32.6	7.89
Kocaman (26)	UC (42)	NA	NA	NA	61.9	NA	NA	NA
Korkmaz (49)	IBD (102)	6.66	22	3.69	97.9	NA	NA	1.9
Kothari (42)	UC (83)	4.75	31.41	2.59	100	NA	20.5	NA
Maharshak (43)	IBD (61)	5.2	23.5	8.74	62	23	28	NA
Nemes (50)	UC (11)	8.1	NA	NA	NA	NA	9	NA
Ozturk (13)	IBD (126)	13	24.4	4.14	94	26	41	13
Papa (44)	IBD (52)	20	42	7.92	46	27	31	33
Petr (58)	CD (21)	2.33	NA	3.2	100	19	52.4	0
Principi (14)	IBD (49)	8.8	25.5	5.42	59	29	29	34
Principi (57)	IBD (23)	2.96	1.5	0.61	100	30.4	13.0	52.2
Roifman (59)	IBD (48)	16.8	28.4	NA	20.8	43.8	16.7	6.3
Theocharidou (45)	IBD (42)	4	NA	8.8	57.1	16.7	45.2	40.5
Theocharidou (51)	IBD (44)	3.5	NA	8.4	47.7	6.8	43.2	45.5
Üstün (27)	IBD (96)	16.1	31.3	6.41	NA	NA	NA	NA
Uysal (46)	IBD (47)	NA	23.4	NA	NA	NA	NA	NA

van Leuven (47)	CD (60)	21	NA	NA	NA	NA	NA	NA
Winderma (60)	IBD (16)	4.4	40.9	5.3	18.8	0	37.5	75
Zanoli (52)	IBD (32)	0.57	18	5.3	100	NA	NA	NA
Zanoli (53)	UC (45)	6.9	NA	NA	64	33	20	22
Zanoli (22)	IBD (167)	1	NA	4	64.1	NA	NA	17.4
Zanoli (54)	IBD (74)	NA	NA	6.45	38	43	43	19
Zanoli (23)	CD (86)	0.82	NA	7.4	50	NA	NA	29.1
Zivkovic (55)	IBD (80)	10.26	NA	7.3	71.3	NA	NA	NA

CRP, C-reactive protein; ESR, erythrocyte sedimentation rate; IM: immunomodulator; UC, ulcerative colitis; CD, Crohn's disease; IBD, inflammatory bowel disease.

Table S3. Outcomes of patients with IBD and Controls in included studies.

Author	Measurement	UC	CD	IBD	Controls
Akdoğan (28)	cIMT (mean±SD, mm)	0.86±0.15	NA	NA	0.55±0.29
	cfPWV (mean±SD, m/s)	8.94±2.98	NA	NA	7.17±1.73
Alkan (29)	cIMT (mean±SD, mm)	0.73±0.1	0.74±0.09	0.74±0.09	0.66±0.06
	cfPWV (mean±SD, m/s)	7.06±0.83	7.19±0.71	7.17±0.92	5.97±0.54
Aloi (30)	cIMT (mean±SD, mm)	0.51±0.10	0.52±0.10	0.52±0.10	0.40±0.09
	Brachial artery FMD (mean±SD, %)	NA	13.70±11.65	NA	31.39±16.61
Aloi (31)	cIMT (mean±SD, mm)	NA	NA	0.52±0.11	0.48±0.06
	aIMT (mean±SD, mm)	0.58±0.07	0.6±0,11	0.60±0.10	0.51±0.05
Andreozzi (56)	Brachial artery FMD (mean±SD, %)	NA	NA	18.4±9.1	26.3±16.5
	RHI (mean±SD)	NA	NA	1.5±0.3	1.6±0.5
Aytac (48)	cfPWV (mean±SD, m/s)	9.28±1.31	9.56±1.41	9.41±1.36	7.59±0.29
Broide (32)	cIMT (mean±SD, mm)	NA	0.51±0.1	NA	0.55±0.07
Bruzzese (33)	cIMT (mean±SD, mm)	0.64±0.19	0.83±0.22	0.68±0.21	0.82±0.2
Caliskan (34)	cIMT (mean±SD, mm)	NA	0.52±0.07	NA	0.4±0.07
Cappello (35)	cIMT (mean±SD, mm)	0.5±0.2	0.5±0.1	0.5±0.2	0.4±0.1
	cfPWV (mean±SD, m/s)	9.1±1.4	8.6±1.3	8.7±1.3	8.0±1.2
	Aortic AIx (AP/PP, %)	15.6±9.4	16.8±11.0	16.4±10.4	11.0±7.8
Dagli (36)	cIMT (mean±SD, mm)	NA	NA	0.74±0.08	0.70±0.05
Ekmen (37)	cIMT (mean±SD, mm)	NA	NA	0.5±0.22	0.4±0.07
Fan (24)	baPWV (mean±SD, m/s)	NA	NA	13.82±2.59	13.45±2.64
Gozel (38)	cIMT (mean±SD, mm)	NA	NA	0.54±0.15	0.41±0.18
Hernández (39)	cIMT (mean±SD, mm)	NA	NA	0.64±0.14	0.60±0.12
Jain (40)	cIMT (mean±SD, mm)	0.68±0.07	NA	NA	0.52±0.04
Kayahan (25)	cIMT (mean±SD, mm)	0.55±0.1	0.49±0.04	0.52±0.08	0.52±0.06

	Brachial artery FMD (mean±SD, %)	10.3±5.4	13.3±6.5	11.9±6.12	18.7±9.2
	Brachial artery NMD (mean±SD, %)	15.2±6.5	18.7±6.8	17±6.8	21.4±9.6
Kim (41)	cIMT (mean±SD, mm)	NA	NA	0.53±0.10	0.54±0.07
Kocaman (26)	Brachial artery FMD (mean±SD, %)	10.91±3.35	NA	NA	18.1±8.1
	Brachial artery NMD (mean±SD, %)	12.75±4.81	NA	NA	31.1±12.6
Korkmaz (49)	cfPWV (mean±SD, m/s)	6.55±1.19	6.31±1.20	6.43±1.20	5.78±0.98
	Aortic AIx (AP/PP, %)	NA	NA	22.75±6.70	21.54±6.59
Kothari (42)	cIMT (mean±SD, mm)	0.55±0.17	NA	NA	0.46±0.13
Maharshak (43)	cIMT (mean±SD, mm)	NA	NA	0.66±0.09	0.64±0.07
Nemes (50)	cfPWV (mean±SD, m/s)	9.17±1.59	NA	NA	7.65±1.42
Ozturk (13)	Brachial artery FMD (mean±SD, %)	9.6±5.1	10.8±4.4	10.1±4.82	15.1±9.7
	cfPWV (mean±SD, m/s)	8.13±1.61	8.16±1.74	8.14±1.66	6.85±0.95
	cIMT (mean±SD, mm)	0.44±0.08	0.40±0.08	0.42±0.08	0.41±0.08
Papa (44)	cIMT (mean±SD, mm)	NA	NA	0.63±0.15	0.53±0.08
Petr (58)	RHI (mean±SD)	NA	1.50±0.55	NA	1.99±1.68
Principi (14)	Brachial artery FMD (mean±SD, %)	5.9±3.5	6.3±2.6	6.1±3.0	8.2±3.4
Principi (57)	Brachial artery FMD (mean±SD, %)	NA	NA	8.67±3.79	13.01±4.97
Roifman (59)	RHI (mean±SD)	1.82±0.10	1.79±0.11	1.80±0.07	2.09±0.08
Theocharidou (45)	cIMT (mean±SD, mm)	0.61±0.06	0.63±0.09	0.62±0.08	0.52±0.06
Theocharidou (51)	cfPWV (mean±SD, m/s)	6.3±1.2	7±1.2	6.8±1.2	6.4±0.9
	Aortic AIx (AP/PP, %)	6.9±14.5	15.2±14.6	12.3±15	13.8±16.2
Üstün (27)	Brachial artery FMD (mean±SD, %)	NA	NA	13.3±7.8	10.5±10.9
	Brachial artery NMD (mean±SD, %)	NA	NA	13.6±8.2	10.0±13.4
	cIMT (mean±SD, mm)	NA	NA	0.52±0.14	0.47±0.10
Uysal (46)	cIMT (mean±SD, mm)	0.54±0.17	0.52±0.15	0.53±0.16	0.51±0.11
van Leuven (47)	cIMT (mean±SD, mm)	NA	0.71±0.17	NA	0.59±0.14

Winderma (60)	RHI (mean±SD)	NA	NA	1.66±0.30	2.02±0.42
Zanoli (52)	cfPWV (mean±SD, m/s)	NA	NA	6.6±1.4	6.0±0.8
	Aortic AIx (AP/PP, %)	NA	NA	7.4±10.5	1.5±15.4
Zanoli (53)	cfPWV (mean±SD, m/s)	8.5±3.2	NA	NA	7.5±2.7
Zanoli (22)	aPWV (mean±SD, m/s)	7.8±2.0	7.9±2.0	7.85±2.0	7.1±1.4
Zanoli (54)	cfPWV (mean±SD, m/s)	7.8±1.7	8.0±1.6	7.90±1.65	7.0±1.1
Zanoli (23)	aPWV (mean±SD, m/s)	NA	7.7±1.8	NA	7.0±1.0
Zivkovic (55)	cfPWV (mean±SD, m/s)	8.83±4.25	7.46±1.98	8.06±3.23	6.42±1.47
	Aortic AIx (AP/PP, %)	15.97±10.81	16.67±11.90	16.36±9.95	10.31±8.19

aIMT, aortic intima-media thickness; cIMT, carotid intima-media thickness; cfPWV, carotid-femoral pulse wave velocity; baPWV, brachial-ankle pulse wave velocity; aPWV, aortic pulse wave velocity; FMD, endothelium dependent flow-mediated dilatation; NMD, nitroglycerin-mediated dilatation; AIx, augmentation index; RHI, reactive hyperemia index.

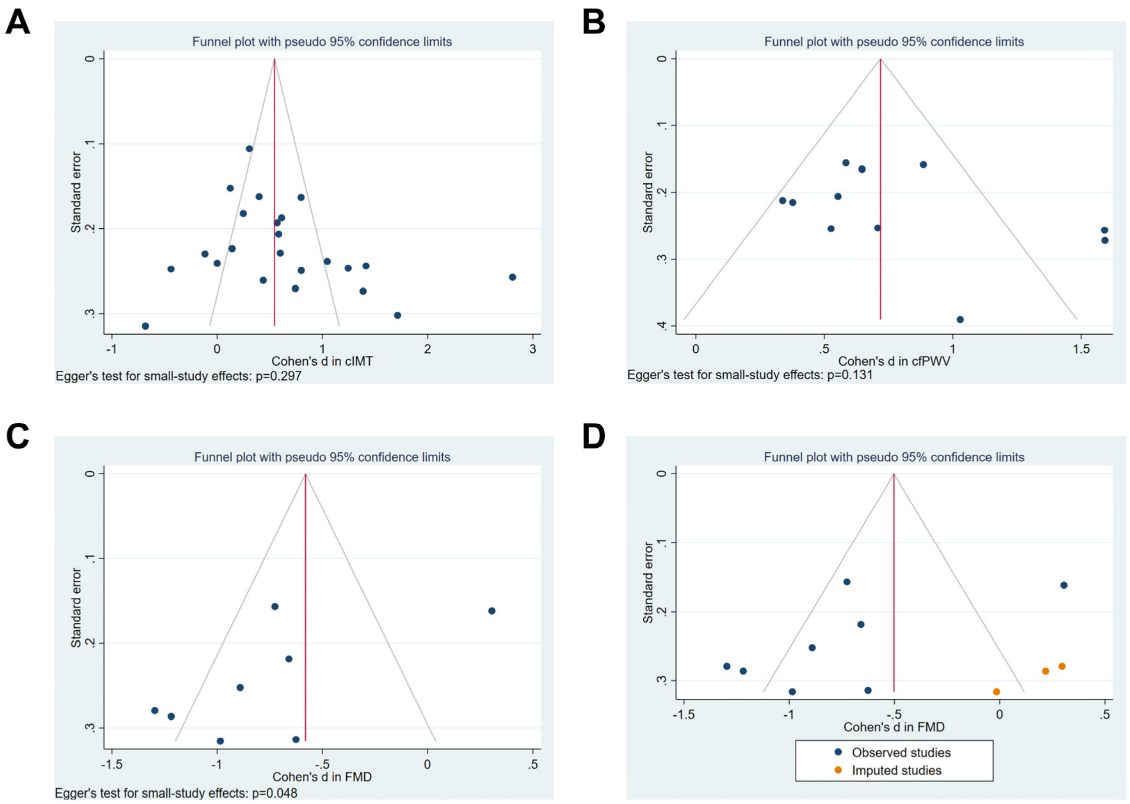

Figure S1. Funnel plot of publication bias of cIMT, cfPWV, and FMD.
